# A Pharmacovigilance Study of Hydroxychloroquine Cardiac Safety Profile: Potential Implication in COVID-19 Mitigation

**DOI:** 10.3390/jcm9061867

**Published:** 2020-06-15

**Authors:** Anand Prakash Singh, Sultan Tousif, Prachi Umbarkar, Hind Lal

**Affiliations:** Division of Cardiovascular Disease, The University of Alabama at Birmingham (UAB), Birmingham, AL 35294-1913, USA; sana80@uab.edu (S.T.); pumbarkar@uabmc.edu (P.U.)

**Keywords:** Hydroxychloroquine, QT prolongation, torsades de pointes, cardiac dysfunction, pharmacovigilance analysis, arrhythmias, COVID-19, cardiovascular adverse events

## Abstract

In light of the favorable outcomes of few small, non-randomized clinical studies, the Food and Drug Administration (FDA) has issued an Emergency Use Authorization (EUA) to Hydroxychloroquine (HCQ) for hospitalized coronavirus disease 2019 (COVID-19) patients. In fact, subsequent clinical studies with COVID-19 and HCQ have reported limited efficacy and poor clinical benefits. Unfortunately, a robust clinical trial for its effectiveness is not feasible at this emergency. Additionally, HCQ was suspected of causing cardiovascular adverse reactions (CV-AEs), but it has never been directly investigated. The objective of this pharmacovigilance analysis was to determine and characterize HCQ-associated cardiovascular adverse events (CV-AEs). We performed a disproportionality analysis of HCQ-associated CV-AEs using the FDA adverse event reporting system (FAERS) database. The FAERS database, comprising more than 11,901,836 datasets and 10,668,655 patient records with drug-adverse reactions, was analyzed. The disproportionality analysis was used to calculate the reporting odds ratios (ROR) with 95% confidence intervals (CI) to predict HCQ-associated CV-AEs. HCQ was associated with higher reporting of right ventricular hypertrophy (ROR: 6.68; 95% CI: 4.02 to 11.17), left ventricular hypertrophy (ROR: 3.81; 95% CI: 2.57 to 5.66), diastolic dysfunction (ROR: 3.54; 95% CI: 2.19 to 5.71), pericarditis (ROR: 3.09; 95% CI: 2.27 to 4.23), torsades de pointes (TdP) (ROR: 3.05; 95% CI: 2.30 to 4.10), congestive cardiomyopathy (ROR: 2.98; 95% CI: 2.01 to 4.42), ejection fraction decreased (ROR: 2.41; 95% CI: 1.80 to 3.22), right ventricular failure (ROR: 2.40; 95% CI: 1.64 to 3.50), atrioventricular block complete (ROR: 2.30; 95% CI: 1.55 to 3.41) and QT prolongation (ROR: 2.09; 95% CI: 1.74 to 2.52). QT prolongation and TdP are most relevant to the COVID-19 treatment regimen of high doses for a comparatively short period and represent the most common HCQ-associated AEs. The patients receiving HCQ are at higher risk of various cardiac AEs, including QT prolongation and TdP. These findings highlight the urgent need for prospective, randomized, controlled studies to assess the risk/benefit ratio of HCQ in the COVID-19 setting before its widespread adoption as therapy.

## 1. Introduction

We are amidst the global coronavirus disease 2019 (COVID-19) pandemic caused by the novel severe acute respiratory syndrome (SARS)-like coronavirus (SARS-CoV-2) [1]. This global pandemic is a serious threat to public health and economic stability. The staggering scale of this disease is now an undisputed fact. This is evident by more than 6.7 million confirmed cases and over 397,388 deaths worldwide [2]. As of the first week of June 2020, more than 2 million infections and 112,549 deaths have been confirmed in the USA alone. Furthermore, these numbers are increasing rapidly. The coronavirus disease illness varies from simple cold symptoms to severe acute respiratory complications. According to a recent report, 80% of patients show symptoms of mild illness, whereas, in 2.3% of cases, the disease is fatal. However, the fatality rate ranges from 8–14.8% in elderly patients (70–79 years) [3]. Once the patient develops severe respiratory complications, and need intensive care and mechanical respiratory assist device (ventilator), the fatality rates goes up to 40–60%.

This extraordinary global public health crisis has challenged the scientist, clinicians, and drug regulatory agencies worldwide to accelerate the development, clinical trial, approval, and implementation of experimental drugs, as well as repurposing of existing therapeutics. Among the numerous therapeutics as potential repurposing candidates for COVID-19, the antimalarial drug hydroxychloroquine (HCQ) has gained significant attention due to favorable outcomes from a few early, small-sized, non-randomized clinical trials [4,5] and evidence from an in vitro experiment [6]. The quick availability and low cost also favor the HCQ. HCQ is an analog of old anti-malarial drug chloroquine (CQ), first synthesized in 1946 by introducing a hydroxyl group (-OH) on CQ. HCQ acts as a weak base and an immunomodulator [7]. Moreover, HCQ is a multipurpose pharmacological drug which is still widely available to treat various kinds of malaria and autoimmune diseases such as systemic lupus erythematosus (SLE) and rheumatoid arthritis (RA) [8]. As a weak lipophilic base, HCQ increases lysosomal pH that interferes with the pH-dependent mechanism of virus replication in target cells. Additionally, HCQ-induced impairment in pH also slows down the immune cells functioning by inhibiting the intracellular processing [9]. Furthermore, being a diprotic week base, HCQ can easily pass plasma membranes and blocks the activity of Toll-like receptor 9 (TLR-9) and Toll-like receptor 7 (TLR-7) to reduce the activation of Dendritic cells (DC) and inflammatory processes [10]. Due to its mechanism of action as a weak base and anti-inflammatory agent, it was easy to put the idea forward that HCQ may be an efficient drug against COVID-19. 

As expected, HCQ showed anti-SARS-CoV activity in vitro by inhibiting the ACE2 receptor-mediated entry of the virus [6]. These findings prompted the initiation of multiple clinical trials worldwide to test the efficacy and safety of HCQ against COVID-19. A ClinicalTrials.gov search on 27 April, 2020 with the term “hydroxychloroquine and COVID”, resulted a list of 140 ongoing clinical trials in various countries (https://clinicaltrials.gov/ct2/results?cond=COVID&term=hydroxychloroquine&cntry=&state=&city=&dist=). The outcome of few early trials with comparatively small sample sizes have shown promising results of HCQ treatment against COVID-19 [4,5,11]. In light of the favorable outcome of these small trials, the Food and Drug Administration (FDA), the primary drug regulatory authority of the US, issued an Emergency Use Authorization (EUA) to HCQ for the treatment of the hospitalized COVID-19 patients. As a result, many USA hospitals had used HCQ as first-line therapy for hospitalized COVID-19 patients despite minimal clinical data supporting its effectiveness and safety. However, in light of the new clinical development regarding the HCQ-associated cardiovascular disease (CVD) safety concern, the focus has certainly shifted away from HCQ for COVID-19 patients. In fact, several recent clinical trials have found increased death in the HCQ-treated arm compared to standard care [4,5]. A 368-COVID-19-patient study, published on a preprint server on 21 April, 2020, reported increased overall mortality in the HCQ-treated group [12]. Furthermore, HCQ alone or with azithromycin did not reduce the need for mechanical ventilation [13]. A double-blinded trial conducted in Brazil found chloroquine (CQ) to be so dangerous at high doses (600 mg CQ twice daily for 10 days) that the trial was halted prematurely due to increased death in CQ-treated patients [14]. The study found that one-quarter of the patients in the CQ arm developed QTc prolongation and fatal arrhythmias. Indeed, there was no benefit regarding efficacy. Taking note of these new clinical developments, on 24 April, 2020, the FDA released a cautionary note against the use of HCQ or CQ for COVID-19 outside of the hospital setting or a clinical trial due to the risk of abnormal heart rhythms such as QT interval prolongation and a dangerously rapid heart rate (ventricular tachycardia) [15]. In this release, the FDA acknowledged that EUA was based upon limited evidence regarding benefit, and its use should be limited to hospitalized patients under careful heart monitoring. Of note, several serious adverse events, including severe allergic reactions and other cardiac problems, have been reported in patients receiving HCQ [3,16]. HCQ can block a crucial potassium channel involved in the maintenance of the heart’s electrical recharging system (ERS) [11,17]. A prolonged QT interval following HCQ use indicated that the drug could interfere with the heart’s rhythm and result in a fatal cardiac adverse event (AE) and torsades de pointes (TdP) in susceptible individuals [16,18]. Currently, several preprints and published papers indicate that about 20–44% of COVID-19 patients develop cardiac complications, including arrhythmias [19,20]. Nevertheless, the rising infection rate of COVID-19 patients, coupled with the widespread use of HCQ, could amplify the incidence of cardiac complications in patients and add to the increasing toll of individuals succumbing to the disease. Furthermore, these risks may increase when HCQ is combined with other medicines known to prolong the QT interval, including the antibiotic azithromycin. 

Taken together, the current data supporting the use of HCQ against COVID-19 include evidence of in vitro activity against SARS-CoV2 and limited clinical research. Hence, there is an urgent need to obtain insights regarding HCQ’s efficacy and safety. Therefore, we performed the first extensive analysis of cardiac adverse reactions of HCQ using the FDA Adverse Event Reporting System (FAERS) pharmacovigilance database and individual case safety reports. We report a high prevalence of right ventricular hypertrophy, left ventricular hypertrophy, diastolic dysfunction, pericarditis, torsades de pointes, congestive cardiomyopathy, ejection fraction decreased, atrioventricular block complete, QT prolongation and right ventricular failure in HCQ-treated patients. Our findings from this extensive analysis of a large database provide a cautionary note for the potential adverse cardiac effects of HCQ, which likely to be further amplified in COVID-19 patients.

## 2. Methods

### 2.1. Study Design and Data Sources

This retrospective analysis has used FAERS pharmacovigilance monitoring database to analyze adverse reactions related to HCQ. We extracted the pertinent data of HCQ from the FAERS database to perform pharmacovigilance disproportionality analysis. This study involves data queries between May 29, 1998 (FDA approval) [21] to December 31, 2019, for HCQ. We adopted the validated pharmacovigilance tool [22,23,24], OpenVigil (version 2.1), to query FAERS database [25,26]. OpenVigil 2.1 has been designed for complete case analysis and is superior for analyses of disproportionality, as it uses cleaned FDA data by removing duplicates and incomplete reports [25]. Data in the event report (also called individual case safety report (ICSR)) include case ID, suspected drug, indication, adverse events, event date, serious outcomes, reporter country, and reporter type. These reports also include the sex (male, female, or unknown) and age of the patient, but do not include their name and date of birth.

### 2.2. Procedures

This observational retrospective study involved queries of adverse events, according to the Medical Dictionary for Regulatory Activities (MedDRA). MedDRA provides a single, standardized international medical terminology that can be used for regulatory communication and evaluation of the data on medicinal products from clinical trials to post-marketing surveillance. It has a hierarchical structure of adverse events terminologies from Standardized MedDRA Queries (SMQs) to Preferred Terms (PTs). We first performed queries for adverse events SMQs for HCQ from 1998 (post-FDA approval) to 2019. Since this study is focused on cardiac safety profile, the following queries were based on PTs for cardiovascular adverse events (CV-AEs). To minimize indication bias, we only performed analysis suspected to be caused by HCQ, not by disease state for which it has been prescribed. The complete list of adverse events data (PTs list, SMQ list and ICSRs) will be shared on request to the corresponding author.

### 2.3. Statistical Analysis

The FAERS database allows for case/non-case analysis, which we have used to compare the risk of CV-AEs associated with HCQ treated cases against CV-AEs reported from other drugs in the entire database. A disproportionality analysis was performed using the reporting odds ratio (ROR) (Supplementary Table S1). The ROR compares the potentially increased risk of adverse events reported with a single drug (e.g., HCQ) with the same adverse events for control group drugs (i.e., the entire database). A specific adverse event with a higher ROR score for a drug suggests a higher chance of detrimental event induction to the patients compared to all other drugs. According to the criteria of Evans [27,28], the ROR signal was considered positive when the number of cases > 3, Chi square values > 4, lower limit of 95% confidence interval (CI) > 1.0, and the ROR value was >2.0. We have performed the analyses using SPSS (version 25.0) and Microsoft excel 2016.

## 3. Results

### 3.1. Cardiovascular Adverse Events Signal Determined for HCQ Using FAERS Database

Emerging clinical data have suggested significant cardiac syndrome during COVID-19 infection [19,29,30]. Therefore, the adverse impact of HCQ during COVID-19 infection, especially cardiac-associated toxicities, is mysterious. To fill the gap between HCQ-mediated cardiotoxicities and how it could affect during COVID-19 treatment, we performed a search query for all SMQs associated with HCQ versus the entire database since its FDA approval from 1998 to 2019. To reduce redundancies and common symptoms for cardiovascular complications, MedDRA has broadly classified it into SMQs. The SMQ analyses of adverse events based on number are summarized in Table 1 with their RORs (95% CI). Using this strategy, we identified nine SMQs with statistically significant RORs for HCQ against the entire database. HCQ was associated with higher reporting of cardiomyopathy, as depicted by elevated ROR (3.04, 95% CI: 2.69 to 3.44). SMQs with higher ROR values are summarized in Supplementary Table S2.

Next, we conducted the queries for PTs, which is a distinct descriptor (single medical concept) for a symptom, sign, and disease diagnosis. The query resulted in a comprehensive list of adverse event PTs for HCQ as compared with the entire database (from 1998–2019). We narrowed down the list with cardiac-related adverse events, shown in Table 2. In the respective duration, the total number of adverse events (AEs) in the FAERS database was 7,687,270. Of those, the AEs related to HCQ were 29,782. As detailed in Table 2, HCQ was associated with higher reporting of right ventricular hypertrophy (RVH) (ROR: 6.68; 95% CI: 4.02 to 11.17), left ventricular hypertrophy (LVH) (ROR: 3.81; 95% CI: 2.57 to 5.66), diastolic dysfunction (DD) (ROR: 3.54; 95% CI: 2.19 to 5.71), pericarditis (ROR: 3.09; 95% CI: 2.27 to 4.23), torsades de pointes (TdP) (ROR: 3.05; 95% CI: 2.30 to 4.10), congestive cardiomyopathy (CC) (ROR: 2.98; 95% CI: 2.01 to 4.42), ejection fraction decreased (EF Dec) (ROR: 2.41; 95% CI: 1.80 to 3.22), right ventricular failure (RVF) (ROR: 2.40; 95% CI: 1.64 to 3.50), atrioventricular block complete (AV Block) (ROR: 2.30; 95% CI: 1.55 to 3.41) and electrocardiogram QT-prolonged (QT prolongation) (ROR: 2.09; 95% CI: 1.74 to 2.52). Other major cardiovascular adverse events like pericardial effusion, atrial fibrillation, and myocardial infarction were not significantly associated with HCQ treatment.

### 3.2. Characteristics of Patients and Outcomes

To better characterize clinical features of HCQ-related cardiovascular AEs, we extracted the individual case safety reports linked with particular AEs. As shown in Table 3, QT prolongation represents the third most common HCQ-associated AE followed by ejection fraction decreased and torsades de pointes. HCQ was the primary suspect (>65%) in the most reports but not in case of pericarditis and right ventricular failure, which represents a higher percentage (>60%) of the accompanying role of HCQ. The maximum peak in reporting of hospitalization (68.18%) was reported in patients having torsades de pointes after receiving HCQ treatment. In contrast to torsades de pointes, QT prolongation had 45.22% hospitalization rate and 36.52% life-threatening outcomes. Right ventricular hypertrophy and Right ventricular failure account for the maximum death percentage (46.66% and 51.85%, respectively) of patients treated with HCQ. The majority of cases in HCQ-associated AEs were reported from the USA, but other countries also have a significant pool of HCQ recipients with these cardiotoxicities. The females having AEs due to HCQ were in preponderance, and patients from all age groups were encountered the AEs. QT prolongation was the only adverse event that occurred in patients under 18 years of age.

**Figure 1:**
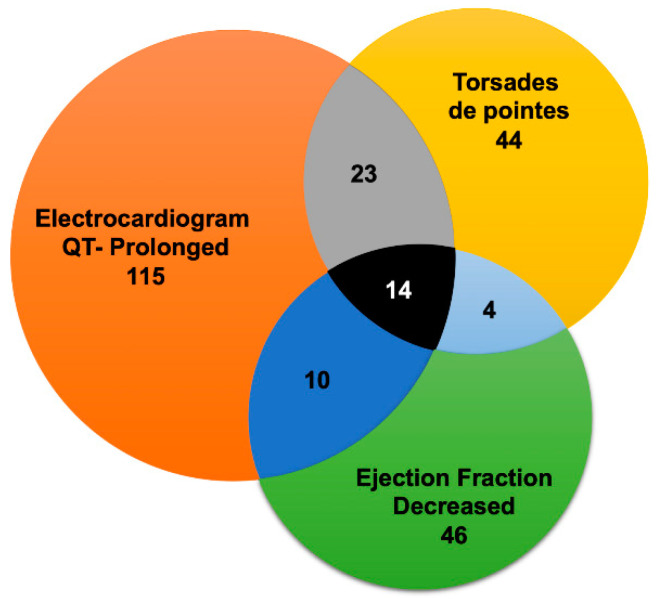
Overlap of cardiovascular adverse events (CV-AEs) associated with Hydroxychloroquine in Food and Drug Administration Adverse Event Reporting System (FAERS) database. Overlap between electrocardiogram QT-prolonged, torsades de pointes, and ejection fraction decreased. Due to diagram limitations, the other CV-AEs overlaps are not displayed. The grey section represents common cases between electrocardiogram QT-prolonged and torsades de pointes, light blue section represents common cases between torsades de pointes and ejection fraction decreased, and dark blue represents common cases between electrocardiogram QT-prolonged and ejection fraction decreased. Black section depicts common cases between electrocardiogram QT-prolonged, torsades de pointes and ejection fraction decreased.

Additionally, our data suggested that drug-associated cardiotoxicities were often related to concurrent contributing AEs (Figure 1). The findings presented herein suggest a direct co-relation of cardiotoxicities with HCQ recipients who had QT prolongation with a shared pool of patients of torsades de pointes and declined ejection fraction (Supplementary Table S3). It is well reported that HCQ falls under the category of QT prolongation medicine [31]. In summary, our findings suggest that HCQ possesses an inherent adverse CVD signal that could further amplify the COVID-19-induced cardiovascular complications. Therefore, we propose detailed cardiac monitoring for COVID-19 patients before and during the HCQ treatment regimen. 

## 4. Discussion

The emergence of the COVID-19 pandemic situation urged the rapid screening of in-hand drugs as a therapeutic option to control the devastating condition. During this endeavor, HCQ has gained a lot of attention, both as a prophylactic as well as a treatment agent. In the same line, we performed the first extensive clinical characterization of adverse cardiovascular reactions associated with HCQ treatment by using the FAERS pharmacovigilance database and an in-depth analysis of individual case safety reports. Our study provides evidence for the existence of HCQ-associated adverse cardiac effects, which were unreported from the last decade due to diagnostic failure and lack of pharmacovigilance analysis. The results from our study suggest a high prevalence of RVH, LVH, DD, pericarditis, torsades de pointes, congestive cardiomyopathy, EF decreased, and RV failure. In our view, among these, QT prolongation and torsades de pointes are most relevant to the COVID-19 treatment regimen of high doses for a comparatively short period. We speculate that the onset of structural remodeling (e.g., hypertrophy) and EF decline may need chronic treatment and are more relevant to HCQ-mediated AEs in systemic lupus erythematosus (SLE) and rheumatoid arthritis (RA) patients. This hypothesis is supported by the recent CloroCOVID-19 trial in which a higher dosage of HCQ analog CQ (600 mg CQ twice daily for 10 days) led to increased QTc prolongation and mortality compared to standard care [14]. Indeed, QT prolongation associated with HCQ treatment has already been labeled as a precaution on recent emergency FDA approval [32] for COVID-19 patients. Thus, our findings are consistent with the recent observation in a double-blinded, randomized clinical trial. As COVID-19 itself adversely affects QT prolongation and arrhythmias, we believe that manifestation of these etiologies will be even more severe to COVID-19 patients treated with HCQ.

Along with QT prolongation and arrhythmias, herein, we also discovered some novel HCQ-mediated CVD adverse effects that have never been reported. We are the first to report HCQ-associated RV hypertrophy, RV failure, and torsades de pointes. QT prolongation and arrhythmias are consistent with the previous reports [31,33,34,35] and meta-analysis. Consistently, we also found that several other cardiac adverse events, including pericardial effusion, atrial fibrillation, and myocardial infarction, are not associated with HCQ in our analysis and served as negative controls. 

Several clinical studies have reported the prevalence of CV complications in COVID-19 patients [36]. One of the commonly reported CV manifestations is arrhythmia. It is believed that the myocardial damage caused by the adverse effects of the viral infection is the primary driver of arrhythmia risk [36]. However, in a clinical trial of 138 hospitalized COVID-19 patients, a remarkable number of arrhythmic cases were observed independent of acute cardiac injury [37]. This disparity suggests the causal role of other factors such as preexisting conduction abnormalities or off-target effects of pharmacological agents that might have enhanced the arrhythmic risk. It is noteworthy that HCQ has previously shown the potential to develop conduction abnormalities [38]. Importantly, it is believed that torsades de pointes (TdP) is a rare event in HCQ-treated patients. It can happen in patients having certain susceptible conditions such as existing electrolyte abnormalities or concomitant treatment of QT-prolonging drugs. However, in our analysis, TdP stood out as an independent, HCQ-associated AE as depicted by a higher ROR value, and >70% of the positive cases where HCQ was the primary suspected drug. 

Our analysis identified new complications linked to the right ventricle due to HCQ treatment. RV hypertrophy has the highest ROR signal (6.68) and mortality (46.66%) among patients treated with HCQ. This suggests that patients receiving HCQ are at higher risk of developing RV hypertrophy. RV failure was associated with a higher mortality rate (51.85%) and significant ROR (2.40). It should be noted here that, in the case of RV failure, HCQ played the role of concomitant medication in 66.66% of cases and still posed a greater risk to patients, which resulted in the highest mortality. Given that cardiac hypertrophic growth is generally an adaptive response of chronic workload or cardiac injury, it is conceivable that HCQ-associated hypertrophic cardiomyopathy may relate to long-term HCQ treatment and/or preexisting CVD comorbidities in the patients.

We also uncover a disproportionate association with diastolic dysfunction for HCQ. Diastolic dysfunction manifests the stiffening of the heart leading to compromised relaxation and ventricular filling defects, thus obscuring the heart failure. Various studies suggested that diastolic dysfunction is usually accompanied by left ventricular hypertrophy [39]. As evidenced by increased ROR for both AEs (LVH:3.81 and DD:3.54) with HCQ treatment, it is highly likely that patients are at an increased risk of developing these AEs. Of note, HCQ was a primary suspect in >85% of cases associated with these AEs and suggested that the development of diastolic dysfunction or LVH does not depend upon preexisting cardiac complications. In addition to these CV-AEs, congestive cardiomyopathy and ejection fraction decreases were also directly related to HCQ treatment. Interestingly, our data also feature pericarditis as a risk factor of HCQ treatment. As HCQ is a potent immunomodulatory drug and COVID-19 patients exhibit a myriad of immune activation [40], the probability of developing pericarditis will also be prevalent. These observations can be helpful in screening vulnerable groups and planning a safe strategy of HCQ treatment regimen in COVID-19 patients. 

The outcomes of recent, small clinical trials have shown that HCQ can shorten the time to clinical recovery (TTCR) and improve pneumonia in COVID-19 patients [5]. Additionally, the potential synergistic benefits of HCQ and azithromycin in viral load reduction among COVID-19 patients are reported in the clinical trial of a small cohort [11]. However, subsequent studies and a recent retrospective analysis of trials verifying the benefits of HCQ vs. HCQ + azithromycin did not show the clinical benefits of HCQ when used as mono or combination therapy [12,13,41]. Strikingly, Magagnoli et al. [12] reported an association of increased overall mortality in patients treated with HCQ alone. Such limited and conflicting data regarding the safety and efficacy of HCQ suggest that extensive randomized controlled studies are needed to assess the risk/benefit ratio of HCQ treatment. While we wait for the outcome of such a study, our extensive analysis provides a comprehensive cardiac safety profile of HCQ that can help to evaluate the safety and efficacy of HCQ treatment among COVID-19 patients.

## 5. Study Limitations

This retrospective study using the FAERS database has several limitations. The baseline cardiac characteristics of patients receiving HCQ are not known. The grade of cardiac AEs and onset of disease after taking medicine are also not reported. In many individual case safety reports (ICSRs), the dosing information is missing, which makes it challenging to predict dose-related consequences. As COVID-19 itself has a substantial adverse effect on CV diseases, including arrhythmias, the same will likely be amplified with HCQ. Considering this fact, findings in our report might be an underestimation of the real scenario of arrhythmia in the COVID-19 plus HCQ setting. This is supported by the CloroCOVID-19 study in which patients got fatal arrhythmias very quickly, even much more than one would expect from the data presented herein. The CV-AE risk prediction by disproportionality analysis with the FAERS pharmacovigilance database has been demonstrated in various studies [22,23,24], but it is of paramount importance that hypotheses generated using the pharmacovigilance database analysis should be validated by prospective studies. The significance of current research is that it will inform clinicians about CV-AEs related to HCQ treatment in a timely manner, which will be highly useful to select the ideal treatment agent for the individual patient suffering from COVID-19.

## 6. Conclusions

The cardiac safety profile of HCQ using pharmacovigilance analysis predicts that the patients receiving HCQ are at higher risk of developing cardiotoxic manifestations. Cardiac AEs, like QT prolongation, TdP, RVH, LVH, DD, etc., pose a higher risk for COVID-19 patients prescribed HCQ. These events must be considered during inpatient care. Finally, before moving to the widespread use of HCQ to mitigate COVID-19, extensive prospective, randomized, placebo-controlled, blinded clinical studies are needed to assess the risk/benefit ratio of HCQ in the COVID-19 setting.

## 7. Translational Perspective

Considering the ongoing worldwide COVID-19 pandemic, and its severe consequences to public health and financial stability, the current findings have obvious clinical implications. Our results suggest that patients receiving HCQ are at higher risk of various cardiotoxicities, including arrhythmias. Recent small clinical trials also support this line of thought. A trial conducted in Brazil found that one-quarter of the patients in the CQ arm (an analog of HCQ) developed QTc prolongation and arrhythmias. COVID-19 itself leads to serious cardiac AEs, therefore, HCQ-mediated cardiac AEs are likely to amplify in COVID-19 and HCQ settings and must be considered in patient care.

## Figures and Tables

**Table 1:** Signal strength for Hydroxychloroquine at the Standardized MedDRA Query (SMQ) level in Food and Drug Administration Adverse Event Reporting System (FAERS) database.

Adverse Event SMQ Term	Number of Events	ROR (95% CI)
Noninfectious diarrhoea	1150	1.51 (1.42–1.69)
Retinal disorders	577	3.9 (3.59–4.24)
Hypertension	544	0.98 (0.90–1.06)
Interstitial lung disease	522	3.27 (3.00–3.57)
Gastrointestinal ulceration	498	1.72 (1.58–188)
Acute renal failure	454	0.73 (0.67–0.80)
Severe cutaneous adverse reactions	388	2.32 (2.10–2.57)
Gastrointestinal nonspecific dysfunction	336	1.35 (1.21–1.51)
Agranulocytosis	307	1.40 (1.25–1.57)
Oropharyngeal infections	293	2.51 (2.24–2.82)
Acute central respiratory depression	270	1.05 (0.93–1.18)
Cardiomyopathy	259	3.04 (2.69–3.44)
Chronic kidney disease	244	0.52 (0.46–0.59)
Anaphylactic reaction	186	1.04 (0.90–1.20)
Vasculitis	176	3.04 (2.62–3.53)
Conjunctival disorders	159	1.79 (1.53–2.09)
Pulmonary hypertension	151	1.64 (1.39–1.92)
Gastrointestinal nonspecific inflammation	145	1.06 (0.90–1.25)
Eosinophilic pneumonia	111	2.80 (2.32–3.37)
Ocular infections	106	2.26 (1.87–2.74)
Biliary tract disorders	95	0.79 (0.65–0.97)
Hyponatraemia	68	0.65 (0.51–0.82)
Pseudomembranous colitis	66	1.48 (1.16–1.89)
Proteinuria	65	1.82 (1.43–2.32)
Corneal disorders	53	1.91 (1.46–2.50)
Haemolytic disorders	40	0.94 (0.69–1.28)
Guillain-Barre syndrome	24	2.42 (1.62–3.61)

Data highlighted in red show significant signals, reporting odds ratio (ROR), and its 95% confidence interval (95% CI) for Standardized MedDRA Queries (SMQs) associated with hydroxychloroquine (HCQ).

**Table 2:** Signal strength of cardiovascular complications of Hydroxychloroquine at the Preferred Terms level in Food and Drug Administration Adverse Event Reporting System (FAERS) database.

Adverse Events (AE)	AEs Due to Hydroxychloroquine	AEs Reported in Full Database (from 1998–2019)	ROR (95% CI)
Right ventricular hypertrophy	15	577	6.68 (4.02–11.17)
Left ventricular hypertrophy	25	1688	3.81 (2.57–5.66)
Diastolic dysfunction	17	1236	3.54 (2.19–5.71)
Pericarditis	40	3324	3.09 (2.27–4.23)
Torsade de pointes	44	3704	3.05 (2.30–4.10)
Congestive cardiomyopathy	25	2160	2.98 (2.01–4.42)
Ejection fraction decreased	46	4921	2.41 (1.80–3.22)
Right ventricular failure	27	2897	2.40 (1.64–3.50)
Atrioventricular block complete	25	2794	2.30 (1.55–3.41)
Electrocardiogram QT-Prolonged *	115	14,148	2.09 (1.74–2.52)
Pericardial Effusion	52	8360	1.6 (1.2–2.1)
Atrial Fibrillation	115	32,481	0.9 (0.8–1.1)
Myocardial Infarction	260	123,095	0.5 (0.5–0.6)

Reporting odds ratio (ROR) and its 95% confidence interval (95% CI) were calculated for cardiovascular adverse drug events (CV-AEs) associated with hydroxychloroquine versus the entire FAERS database. The significant signals for CV-AEs are highlighted in red. * Electrocardiogram-QT prolonged has been referred as QT Prolongation throughout the manuscript.

**Table 3:** Characteristics of adverse events reports related to hydroxychloroquine from 1998 to 2019.

Type of Adverse Event	Right Ventricular Hypertrophy (%)	Torsades de Pointes (%)	Pericarditis (%)	Electrocardiogram QT-Prolonged (%)	Left Ventricular Hypertrophy (%)	Diastolic Dysfunction (%)	Congestive Cardiomyopathy (%)	Ejection Fraction Decreased (%)	RV Failure (%)	AV Block Complete (%)
**Number of cases**	15	44	40	115	25	17	25	46	27	25
**Indication**									
**SLE**	5 (33.33)	10 (22.72)	10 (25)	27 (21.73)	15 (60)	7 (41.17)	7 (28)	14 (30.43)	2 (7.40)	8 (32)
**RA**	2 (13.33)	6 (13.63)	2 (5)	12 (10.43)	3 (12)	5 (29.41)	7 (28)	4 (8.69)	2 (7.40)	4 (16)
**Other/Unknown**	8 (53.33)	28 (63.63)	28 (70)	76 (66.08)	7 (28)	5 (29.41)	11 (44)	28 (60.86)	23 (85.18)	13 (52)
**Gender**									
**Male**	3 (20)	6 (13.63)	12 (30)	13 (11.30)	21 (84)	2 (11.76)	5 (20)	11 (23.91)	0 (0)	2 (8)
**Female**	11 (73)	33 (75)	25 (62.5)	81 (70.43)	4 (16)	15 (88.24)	19 (76)	34 (73.91)	23 (85.18)	21 (84)
**Unknown**	1 (6.66)	5 (11.36)	3 (7.5)	21 (18.26)	0 (0)	0 (0)	1 (4)	1 (2.17)	4 (14.81)	2 (8)
**Age (Year)**									
**<18**	0 (0)	0 (0)	0 (0)	8 (6.9)	0 (0)	0 (0)	0 (0)	0 (0)	0 (0)	0 (0)
**18–44**	0 (0)	18 (40.90)	7 (17.50)	53 (46.08)	1 (4)	0 (0)	5 (20)	15 (32.60)	8 (29.62)	1 (4)
**45–64**	6 (40)	8 (18.18)	17 (42.5)	20 (17.39)	13 (52)	8 (47.06)	9 (36)	17 (36.95)	5 (18.51)	11 (44)
**>65**	5 (33.33)	11 (25)	1 (2.5)	9 (7.82)	11 (44)	7 (41.18)	6 (24)	12 (26.08)	8 (29.62)	6 (24)
**Unknown**	4 (26.66)	7 (15.90)	15 (37.5)	25 (21.73)	1 (4)	2 (11.76)	5 (20)	2 (4.34)	6 (22.22)	7 (28)
**HCQ role code**									
**Primary Suspect**	11 (73)	32 (72.72)	8 (20)	84 (73.04)	24 (96)	14 (82.35)	17 (68)	37 (80.43)	4 (14.81)	19 (76)
**Secondary Suspect**	2 (13.33)	8 (18.18)	7 (17.5)	8 (6.95)	0 (0)	1 (5.88)	0 (0)	3 (6.52)	5 (18.51)	3 (12)
**Concomitant**	2 (13.33)	4 (9.09)	25 (62.5)	22 (19.13)	1 (4)	2 (11.76)	8 (32)	6 (13.04)	18 (66.66)	3 (12)
**Interacting**	0 (0)	0 (0)	0 (0)	1 (0.86)	0 (0)	0 (0)	0 (0)	0 (0)	0 (0)	0 (0)
**Serious outcomes**									
**Hospitalization**	1 (6.66)	30 (68.18)	25 (62.5)	52 (45.22)	5 (20)	7 (41.18)	6 (24)	19 (41.30)	11 (40.74)	9 (36)
**Life-threatening**	1 (6.66)	9 (20.45)	0 (0)	42 (36.52)	1 (4)	0 (0)	3 (12)	5 (10.86)	0 (0)	2 (8)
**Death**	7 (46.66)	5 (11.36)	1 (2.5)	0 (0)	6 (24)	3 (17.65)	5 (20)	12 (26.08)	14 (51.85)	4 (16)
**Other**	6 (40)	0 (0)	14 (35)	21 (18.26)	13 (52)	7 (41.18)	11 (44)	10 (21.73)	2 (7.40)	10 (40)
**Reporter Country**									
**USA**	8 (53.33)	28 (63.63)	22 (55)	66 (57.39)	5 (20)	5 (29.41)	4 (16)	25 (54.34)	13 (48.14)	8 (32)
**Canada**	5 (33.33)	0 (0)	1 (2.5)	3 (2.60)	15 (60)	7 (41.18)	0 (0)	2 (4.34)	1 (3.70)	3 (12)
**Other Countries**	2 (13.33)	15 (34.09)	12 (30)	41 (35.65)	4 (16)	5 (29.41)	20 (80)	13 (28.26)	8 (29.62)	10 (40)
**Not specified**	0 (0)	1 (2.27)	5 (12.5)	5 (4.34)	1 (4)	0 (0)	1 (4)	6 (13.04)	5 (18.51)	4 (16)

Data last accessed on 24 April, 2020. Abbreviations: AV: Atrioventricular, USA: United States of America, HCQ: Hydroxychloroquine, SLE: Systemic lupus erythematosus, RA: Rheumatoid arthritis.

## Supplementary Materials

The following are available online at https://www.mdpi.com/2077-0383/9/6/1867/s1, Table S1: Calculation of reporting odds ratio (ROR) with hydroxychloroquine versus all other drugs in FAERS database, Table S2: Disproportionality signal analysis of adverse events by reporting odds ratio (ROR) related to hydroxychloroquine at Standardized MedDRA Query (SMQ) level (top 25 listed by higher ROR value), Table S3: Common number of patients in each group of adverse reactions.

## References

[1] World Health Organization Who Director-General’s Opening Remarks at the Media Briefing on Covid-19–11 March 2020. https://www.who.int/dg/speeches/detail/who-director-general-s-opening-remarks-at-the-media-briefing-on-covid-19---11-march-2020.

[2] World Health Organization (2020). Coronavirus Disease (Covid-2019) Situation Reports.

[3] Wu Z., McGoogan J.M. (2020). Characteristics of and important lessons from the coronavirus disease 2019 (COVID-19) outbreak in China: Summary of a report of 72314 cases from the chinese center for disease control and prevention. JAMA.

[4] Abena P.M., Decloedt E.H., Bottieau E., Suleman F., Adejumo P., Sam-Agudu N.A., Muyembe TamFum J.-J., Seydi M., Eholie S.P., Mills E.J. (2020). Chloroquine and hydroxychloroquine for the prevention or treatment of novel coronavirus disease (COVID-19) in Africa: Caution for inappropriate off-label use in healthcare settings. Am. J. Trop. Med. Hyg..

[5] Chen Z., Hu J., Zhang Z., Jiang S., Han S., Yan D., Zhuang R., Hu B., Zhang Z. (2020). Efficacy of hydroxychloroquine in patients with COVID-19: Results of a randomized clinical trial. medRxiv.

[6] Liu J., Cao R., Xu M., Wang X., Zhang H., Hu H., Li Y., Hu Z., Zhong W., Wang M. (2020). Hydroxychloroquine, a less toxic derivative of chloroquine, is effective in inhibiting SARS-CoV-2 infection in vitro. Cell Discov..

[7] McChesney E.W. (1983). Animal toxicity and pharmacokinetics of hydroxychloroquine sulfate. Am. J. Med..

[8] Drugs.com (2020). Hydroxychloroquine Sulfate Monograph for Professionals. https://www.drugs.com/monograph/hydroxychloroquine-sulfate.html.

[9] Meyerowitz E.A., Vannier A.G.L., Friesen M.G.N., Schoenfeld S., Gelfand J.A., Callahan M.V., Kim A.Y., Reeves P.M., Poznansky M.C. (2020). Rethinking the role of hydroxychloroquine in the treatment of COVID-19. FASEB J..

[10] Sacre K., Criswell L.A., McCune J.M. (2012). Hydroxychloroquine is associated with impaired interferon-alpha and tumor necrosis factor-alpha production by plasmacytoid dendritic cells in systemic lupus erythematosus. Arthritis Res..

[11] Gautret P., Lagier J.C., Parola P., Hoang V.T., Meddeb L., Mailhe M., Doudier B., Courjon J., Giordanengo V., Vieira V.E. (2020). Hydroxychloroquine and azithromycin as a treatment of COVID-19: Results of an open-label non-randomized clinical trial. Int. J. Antimicrob. Agents.

[12] Magagnoli J., Narendran S., Pereira F., Cummings T., Hardin J.W., Sutton S.S., Ambati J. (2020). Outcomes of hydroxychloroquine usage in United States veterans hospitalized with Covid-19. medRxiv.

[13] Molina J.M., Delaugerre C., Le Goff J., Mela-Lima B., Ponscarme D., Goldwirt L., de Castro N. (2020). No evidence of rapid antiviral clearance or clinical benefit with the combination of hydroxychloroquine and azithromycin in patients with severe COVID-19 infection. Med. Mal. Infect..

[14] Borba M.G.S., Val F.F.A., Sampaio V.S., Alexandre M.A.A., Melo G.C., Brito M., Mourao M.P.G., Brito-Sousa J.D., Baia-da-Silva D., Guerra M.V.F. (2020). Effect of high vs. low doses of chloroquine diphosphate as adjunctive therapy for patients hospitalized with severe acute respiratory syndrome coronavirus 2 (SARS-CoV-2) infection: A randomized clinical trial. JAMA Netw. Open.

[15] U.S. Food & Drug Administration (2020). FDA Cautions against Use of Hydroxychloroquine or Chloroquine for Covid-19 Outside of the Hospital Setting or a Clinical Trial due to Risk of Heart Rhythm Problems. https://www.fda.gov/media/137250/download.

[16] Mayo Clinic (2020). Guidance on Patients at Risk of Drug-Induced Sudden Cardiac Death from Off-Label Covid-19 Treatments. https://newsnetwork.mayoclinic.org/discussion/mayo-clinic-provides-urgent-guidance-approach-to-identify-patients-at-risk-of-drug-induced-sudden-cardiac-death-from-use-of-off-label-covid-19-treatments/.

[17] Chen C.Y., Wang F.L., Lin C.C. (2006). Chronic hydroxychloroquine use associated with QT prolongation and refractory ventricular arrhythmia. Clin. Toxicol. (Phila).

[18] Kapoor A., Pandurangi U., Arora V., Gupta A., Jaswal A., Nabar A., Naik A., Naik N., Namboodiri N., Vora A. (2020). Cardiovascular risks of hydroxychloroquine in treatment and prophylaxis of COVID-19 patients: A scientific statement from the Indian Heart Rhythm Society. Indian Pacing Electrophysiol. J..

[19] Shi S., Qin M., Shen B., Cai Y., Liu T., Yang F., Gong W., Liu X., Liang J., Zhao Q. (2020). Association of cardiac injury with mortality in hospitalized patients with COVID-19 in Wuhan, China. JAMA Cardiol..

[20] Klok F.A., Kruip M., van der Meer N.J.M., Arbous M.S., Gommers D., Kant K.M., Kaptein F.H.J., van Paassen J., Stals M.A.M., Huisman M.V. (2020). Incidence of thrombotic complications in critically ill ICU patients with COVID-19. Thromb. Res..

[21] U.S. Food & Drug Administration (1998). Hydroxychloroquine Sulfate Drugs@FDA: FDA-Approved Drugs. https://www.accessdata.fda.gov/scripts/cder/daf/index.cfm?event=overview.process&ApplNo=040274.

[22] Meng L., Huang J., Jia Y., Huang H., Qiu F., Sun S. (2019). Assessing fluoroquinolone-associated aortic aneurysm and dissection: Data mining of the public version of the FDA adverse event reporting system. Int. J. Clin. Pract..

[23] Huang J., Meng L., Yang B., Sun S., Luo Z., Chen H. (2020). Safety profile of epidermal growth factor receptor tyrosine kinase inhibitors: A disproportionality analysis of FDA adverse event reporting system. Sci. Rep..

[24] Anand K., Ensor J., Trachtenberg B., Bernicker E.H. (2019). Osimertinib-induced cardiotoxicity: A retrospective review of the FDA Adverse Events Reporting System (FAERS). JACC Cardiooncol..

[25] Bohm R., von Hehn L., Herdegen T., Klein H.J., Bruhn O., Petri H., Hocker J. (2016). OpenVigil FDA—Inspection of U.S. American adverse drug events pharmacovigilance data and novel clinical applications. PLoS ONE.

[26] Bohm R., Hocker J., Cascorbi I., Herdegen T. (2012). OpenVigil—Free eyeballs on AERS pharmacovigilance data. Nat. Biotechnol..

[27] Bate A., Evans S.J. (2009). Quantitative signal detection using spontaneous ADR reporting. Pharm. Drug Saf..

[28] Evans S.J., Waller P.C., Davis S. (2001). Use of proportional reporting ratios (PRRs) for signal generation from spontaneous adverse drug reaction reports. Pharm. Drug Saf..

[29] Inciardi R.M., Lupi L., Zaccone G., Italia L., Raffo M., Tomasoni D., Cani D.S., Cerini M., Farina D., Gavazzi E. (2020). Cardiac involvement in a patient with coronavirus disease 2019 (COVID-19). JAMA Cardiol..

[30] Guo T., Fan Y., Chen M., Wu X., Zhang L., He T., Wang H., Wan J., Wang X., Lu Z. (2020). Cardiovascular implications of fatal outcomes of patients with coronavirus disease 2019 (COVID-19). JAMA Cardiol..

[31] Chatre C., Roubille F., Vernhet H., Jorgensen C., Pers Y.M. (2018). Cardiac complications attributed to chloroquine and hydroxychloroquine: A systematic review of the literature. Drug Saf..

[32] U.S. Food & Drug Administration (2020). EUA Hydroxychloroquine Sulfate Health Care Provider Fact Sheet. https://www.fda.gov/media/136537/download.

[33] OHDSI—Observational Health Data Sciences and Informatics (2020). Hydroxychloroquine Safety and Potential Efficacy as an Antiviral Prophylaxis in Light of Potential Wide-Spread Use in Covid-19: A Multinational, Large-Scale Network Cohort and Self-Controlled Case Series Study. http://www.encepp.eu/encepp/viewResource.htm%3Fid=34498.

[34] Chorin E., Wadhwani L., Magnani S., Dai M., Shulman E., Nadeau-Routhier C., Knotts R., Bar-Cohen R., Kogan E., Barbhaiya C. (2020). QT interval prolongation and torsade de pointes in patients with COVID-19 treated with hydroxychloroquine/azithromycin. Heart Rhythm.

[35] Sarayani A., Cicali B., Henriksen C.H., Brown J.D. (2020). Safety signals for QT prolongation or torsades de pointes associated with azithromycin with or without chloroquine or hydroxychloroquine. Res. Soc. Adm. Pharm..

[36] Driggin E., Madhavan M.V., Bikdeli B., Chuich T., Laracy J., Bondi-Zoccai G., Brown T.S., Nigoghossian C., Zidar D.A., Haythe J. (2020). Cardiovascular considerations for patients, health care workers, and health systems during the coronavirus disease 2019 (COVID-19) pandemic. J. Am. Coll. Cardiol..

[37] Wang D., Hu B., Hu C., Zhu F., Liu X., Zhang J., Wang B., Xiang H., Cheng Z., Xiong Y. (2020). Clinical characteristics of 138 hospitalized patients with 2019 novel coronavirus-infected pneumonia in Wuhan, China. JAMA.

[38] Page R.L., O’Bryant C.L., Cheng D., Dow T.J., Ky B., Stein C.M., Spencer A.P., Trupp R.J., Lindenfeld J., American Heart Association Clinical Pharmacology (2016). Drugs that may cause or exacerbate heart failure: A scientific statement from the American heart association. Circulation.

[39] Kitzman D.W. (2000). Diastolic dysfunction in the elderly. Genesis and diagnostic and therapeutic implications. Cardiol. Clin..

[40] Hua A., O’Gallagher K., Sado D., Byrne J. (2020). Life-threatening cardiac tamponade complicating myo-pericarditis in COVID-19. Eur. Heart J..

[41] Mahevas M., Tran V.-T., Roumier M., Chabrol A., Paule R., Guillaud C., Gallien S., Lepeule R., Szwebel T.-A., Lescure X. (2020). No evidence of clinical efficacy of hydroxychloroquine in patients hospitalized for COVID-19 infection with oxygen requirement: Results of a study using routinely collected data to emulate a target trial. medRxiv.

