# Supplementary material for: A Pharmacovigilance Study of Hydroxychloroquine Cardiac Safety Profile: Potential Implication in COVID-19 Mitigation
Source: J Clin Med. 2020 Jun 15;9(6):1867. doi: 10.3390/jcm9061867 (PMC7355808; doi:10.3390/jcm9061867)
Supplement: Supplementary file 1 [file jcm-09-01867-s001.pdf]

Supplementary Table S1: Calculation of reporting odds ratio (ROR) with hydroxychloroquine versus all other drugs in FAERS database.

	Hydroxychloroquine	All other drugs in FAERS
Reports of AE of interest	A	B
All other events	C	D

ROR= (AxD)/(BxC), 95%CI $= e^{\ln(ROR) \pm 1.96\sqrt{(1/A+1/B+1/C+1/D)}}$.

Supplementary Table S2: Disproportionality signal analysis of adverse events by reporting odds ratio (ROR) related to hydroxychloroquine at Standardized MedDRA Query (SMQ) level (top 25 listed by higher ROR value).

Adverse event SMQ Terms	Number of Events	ROR	95% CI Lower	95% CI Upper
Ovarian tumours of unspecified malignancy	49	33.56	24.91	45.21
Noninfectious encephalitis	84	6.82	5.49	8.47
Breast tumours of unspecified malignancy	8	3.99	1.99	8.03
Retinal disorders	577	3.90	3.59	4.24
Interstitial lung disease	522	3.27	3.00	3.57
Congenital and neonatal arrhythmias	12	3.20	1.81	5.66
Glaucoma	183	3.06	2.65	3.55
Cardiomyopathy	259	3.04	2.69	3.44
Vasculitis	176	3.04	2.62	3.53
Haemodynamic oedema effusions and fluid overload	2871	2.86	2.75	2.97
Eosinophilic pneumonia	111	2.80	2.32	3.37
Lacrimal disorders	217	2.78	2.43	3.18
Noninfectious meningitis	106	2.59	2.14	3.14
Osteoporosis/osteopenia	223	2.55	2.23	2.91
Oropharyngeal infections	293	2.51	2.24	2.82
Lens disorders	196	2.46	2.14	2.83
Guillain-Barre syndrome	24	2.42	1.62	3.61
Skin malignant tumours	215	2.41	2.11	2.76
Scleral disorders	15	2.35	1.41	3.91
Severe cutaneous adverse reactions	388	2.32	2.10	2.57
Premalignant disorders general conditions and other site specific disorders	32	2.31	1.63	3.27
Ocular infections	106	2.26	1.87	2.74
Malignant lymphomas	176	2.21	1.90	2.56
Oropharyngeal neoplasms	36	2.16	1.56	3.00
Uterine and fallopian tube malignant tumours	35	2.02	1.45	2.81

Supplementary Table S3: Common number of patients in each group of adverse reactions.

RVH vs	Common number of patients	TdP vs	Common number of patients
TdP	0	RVH	0
Pericarditis	0	Pericarditis	0
QT Prolongation	0	QT Prolongation	23
LVH	5	LVH	1
DD	4	DD	0
CC	0	Congestive Card.	0
EF Dec	4	EF Dec	4
RV Failure	0	RV Failure	0
AV Block comp.	1	AV Block comp.	0

Pericarditis vs	Common number of patients	QT Prolongation vs	Common number of patients
TdP	0	TdP	23
RVH	0	Pericarditis	0
QT Prolongation	0	RVH	0
LVH	0	LVH	2
DD	0	DD	0
Congestive Card.	0	Congestive Card.	0
EF Dec	0	EF Dec	10
RV Failure	0	RV Failure	0
AV Block comp.	0	AV Block comp.	0

DD vs	Common number of patients	LVH vs	Common number of patients
TdP	0	TdP	1
Pericarditis	0	Pericarditis	0
QT Prolongation	0	QT Prolongation	2
LVH	10	RVH	5
RVH	4	DD	10
Congestive Card.	0	Congestive Card.	0
EF Dec	2	EF Dec	8
RV Failure	1	RV Failure	0
AV Block comp.	3	AV Block comp.	2

Congestive Card. vs	Common number of patients	EF Dec vs	Common number of patients
TdP	0	TdP	4
Pericarditis	0	Pericarditis	0

QT Prolongation	0	QT Prolongation	10
LVH	0	LVH	8
DD	0	DD	2
RVH	0	Congestive Card.	2
EF Dec	2	RVH	4
RV Failure	0	RV Failure	0
AV Block comp.	0	AV Block comp.	3

RV Failure vs	Common number of patients	AV Block comp. vs	Common number of patients
TdP	0	TdP	0
Pericarditis	0	Pericarditis	0
QT Prolongation	0	QT Prolongation	0
LVH	0	LVH	2
DD	1	DD	3
Congestive Card.	0	Congestive Card.	0
EF Dec	0	EF Dec	3
RVH	0	RV Failure	0
AV Block comp.	0	RVH	1

Abbreviations: vs: versus, RVH: right ventricular hypertrophy, Tdp: torsades de pointes, LVH: left ventricular hypertrophy, DD: diastolic dysfunction, Congestive Card.: congestive cardiomyopathy, EF Dec: ejection fraction decreased, RV Failure: right ventricular failure, AV Block comp.: Atrioventricular block complete.
